# Wuwei Jianpi San Improves Growth Performance and Immune Status in Yaks Through Modulation of Rumen Microbiota and Host Metabolism

**DOI:** 10.3390/ani16101539

**Published:** 2026-05-18

**Authors:** Ke Zhou, Hongmei Shi, Xiangying Kong, Weidong Ma, Jianguo Kang, Haizhong Che, Yongli Hua

**Affiliations:** 1 College of Veterinary Medicine, Gansu Agricultural University, Lanzhou 730070, China; 107332201047@st.gsau.edu.cn (K.Z.); 1073325140028@st.gsau.edu.cn (J.K.); 2 Gansu Gannan Animal Husbandry and Veterinary Workstation, Gannan 747000, China; shmgzygd@163.com; 3 Qinghai Haibei Animal Husbandry and Veterinary Science Research Institute, Haibei 812200, China; 13897309088@163.com (X.K.); chz878787@163.com (H.C.); 4 Shaanxi Agricultural and Animal Husbandry Breeding Farm, Baoji 722200, China; 13689271488@163.com

**Keywords:** Wuwei Jianpi San, yak, rumen microbiota, cytokines, tryptophan metabolism

## Abstract

To investigate the effects of Wuwei Jianpi San (WJPS), a Chinese herbal compound feed additive, on rumen microecology, host metabolism, and immune function in healthy yaks (*Bos grunniens*), and to determine the optimal supplementation level, 32 yaks with similar initial body weight were randomly assigned to four groups: a control group and three groups receiving 0.5%, 1.0%, or 2.0% WJPS for 90 days. Growth performance, hematological indices, serum antioxidant and immune parameters, tryptophan metabolites, ruminal short-chain fatty acids (SCFAs), and rumen microbiota were analyzed. WJPS supplementation improved growth performance, as shown by a reduced feed-to-gain ratio in all treated groups and tended to increase average daily gain in the 2.0% group. It also enhanced hematological, antioxidant, and immune status, evidenced by increased white blood cell (WBC) and lymphocyte (Lym) counts and elevated interleukin-2 (IL-2), immunoglobulin G (IgG), and superoxide dismutase (SOD) levels. Moreover, 2.0% WJPS increased total SCFAs, acetate, and n-butyrate, while WJPS reduced kynurenine pathway metabolites, including kynurenine, 3-hydroxykynurenine, and quinolinic acid. Metagenomic analysis showed that WJPS tended to shape rumen microbial composition by increasing Bacillota and decreasing Bacteroidota, and these microbial changes were associated with host immune indices and tryptophan metabolism. Overall, 2.0% WJPS showed the best comprehensive effect.

## Simple Summary

Wuwei Jianpi San (WJPS) is a traditional Chinese herbal compound that may help improve animal health when used as a feed additive. In this study, we evaluated whether dietary WJPS could improve growth, rumen function, metabolism, and immunity in healthy yaks, and we compared different supplementation levels. Thirty-two yaks (*Bos grunniens*) were fed diets containing 0%, 0.5%, 1.0%, or 2.0% WJPS for 90 days. The results showed that WJPS improved growth performance, reduced feed conversion ratio, enhanced blood and immune indicators, increased antioxidant capacity, and promoted rumen short-chain fatty acid production. It also helped maintain rumen epithelial integrity and reduced harmful metabolites related to tryptophan metabolism. In addition, WJPS tended to modulate the rumen microbial community in a beneficial way, and these microbial changes were closely associated with host physiological status. Among the tested levels, 2.0% WJPS showed the best overall effect. These findings suggest that WJPS has potential as a natural feed additive for improving health and productivity in yak production.

## 1. Introduction

The yak (*Bos grunniens*) is a characteristic ruminant species of alpine pastoral regions and plays an essential role in local ecological stability and sustainable livestock production. As the primary site of feed fermentation and nutrient transformation, the rumen is central to nutrient utilization, immune competence, and overall health in yaks [1,2,3,4]. Under high-altitude conditions, particularly during winter when forage supply and quality decline markedly, yaks are highly susceptible to nutritional insufficiency and environmental stress, which may disrupt rumen microbial homeostasis [5,6]. Such dysbiosis can impair immune function, disturb inflammatory balance, and ultimately reduce production efficiency [7,8]. Therefore, the development of natural and effective feed additives that improve yak health through modulation of rumen microbiota and its associated metabolic processes is of considerable importance for sustainable yak production.

Chinese herbal compound preparations have attracted increasing attention as natural feed additives in livestock because of their potential to modulate microbial communities and improve digestive efficiency, antioxidant status, and immune function [9]. For example, traditional Chinese medicine (TCM) compounds have been shown to alter rumen fermentation and microbial populations, while *Astragalus membranaceus* and *Codonopsis pilosula* have been reported to improve nutrient utilization, intestinal health, microbial composition, and host immune status in livestock [10,11].

Wuwei Jianpi San (WJPS) is a traditional Chinese veterinary herbal formula composed of *Codonopsis pilosula*, *Astragalus membranaceus*, *Crataegus pinnatifida*, *Citrus reticulata*, and *Massa Medicata Fermentata*. WJPS comprises Codonopsis pilosula and Astragalus membranaceus to reinforce qi and invigorate the spleen, Crataegus pinnatifida and Massa Medicata Fermentata to promote digestion and resolve food stagnation, and Citrus reticulata to regulate “qi” and harmonize the stomach. “Jianpi” (invigorating the spleen) in TCM corresponds to enhancing digestive and absorptive function in ruminants, thereby supporting rumen fermentation efficiency and nutrient utilization. For example, fermented medicinal dregs of Wuwei Jianpi granules improved average daily gain, antioxidant capacity, and microbial diversity in finishing sheep [12]. However, current evidence remains limited and fragmented, and the effects of WJPS in yaks have not been systematically clarified. In particular, it is still unclear whether WJPS can improve growth performance and host physiological status in yaks through coordinated regulation of rumen microbial composition, fermentation characteristics, and microbiota-associated metabolic and immune processes. Tryptophan metabolism is a critical hub linking immune activation, inflammatory status, and energy homeostasis, making it a key marker of immune-metabolic health in yaks under high-altitude stress. Moreover, the potential relationships among rumen microbial remodeling, short-chain fatty acid production, tryptophan metabolism, and host antioxidant and immune responses under plateau conditions remain poorly understood.

Therefore, this study investigated the effects of different dietary levels of WJPS on growth performance, rumen fermentation, antioxidant and immune indices, tryptophan metabolism, and rumen microbial community structure in healthy yaks. By integrating fermentation characteristics, serum physiological indicators, and rumen metagenomic analysis, this study aimed to clarify the dose-dependent effects of WJPS, identify the optimal supplementation level, and explore the potential associations between key rumen microorganisms and host metabolic and immune traits. The findings of this study provide experimental evidence for the application of WJPS as a natural feed additive in plateau yak production and may further improve current understanding of how Chinese herbal compound preparations regulate rumen microecology and host physiological homeostasis in ruminants.

## 2. Materials and Methods

### 2.1. Experimental Design and Materials

This experiment was conducted at Huangcheng Laomuren Animal Husbandry Professional Cooperative, Menyuan County, Haibei Prefecture, Qinghai Province, China. All animal procedures were carried out in accordance with the relevant guidelines for the care and use of experimental animals and were approved by the Animal Ethics Committee of Gansu Agricultural University (Approval No. GSAU-Eth-VMC-2024-054).

A total of 32 healthy yaks with similar initial body weight (150 ± 40 kg) were enrolled in the experiment. Before allocation, all animals were ranked by body weight from lowest to highest and assigned to four groups using a zigzag method to ensure balanced body weight distribution across treatments. The yaks were randomly assigned to four dietary treatment groups, with eight animals per group: a control group (CON) fed the basal concentrate diet without WJPS, a low-dose group supplemented with 0.5% WJPS (WJPS-L), a medium-dose group supplemented with 1.0% WJPS (WJPS-M), and a high-dose group supplemented with 2.0% WJPS (WJPS-H).

The WJPS used in this study was a Chinese herbal compound composed of *Codonopsis pilosula*, *Astragalus membranaceus*, *Crataegus pinnatifida*, *Massa Medicata Fermentata*, and *Citrus reticulata* at a ratio of 2:2:1:1:1. All herbal materials were purchased from the Huanghe Chinese Herbal Medicine Market (Lanzhou, China). Yaks were fed oat hay as roughage, with a forage-to-concentrate ratio of 40:60. The daily feeding amount was 2 kg of concentrate and 3 kg of oat hay per head. The nutritional composition of the basal concentrate diet is shown in Table 1, and the diets were formulated according to the Nutrient Requirements of Yaks (DB63/T 2360-2024, Qinghai Provincial Local Standard [13]) to ensure consistent macro- and micronutrient levels across all treatment groups.

The experiment lasted for 90 days, including a 14-day adaptation period and a 76-day formal feeding period. All yaks were housed individually under standardized management conditions, with separate feed troughs to allow measurement of feed refusals. Animals were maintained under a 12 h light/12 h dark cycle, with mechanical ventilation providing six air changes per hour and a stocking density of 2.5 m^2^ per head. Before the start of the trial, the barn and pens were thoroughly cleaned and disinfected. During the experiment, yaks were fed twice daily at 08:00 and 18:00, with WJPS powder thoroughly mixed into the concentrate portion of the total mixed ration to ensure uniform intake, and free access to clean drinking water and mineral salt licks throughout the study.

### 2.2. Sample Collection

To evaluate growth performance, body weight and feed intake were recorded for all 32 yaks throughout the experiment, and average daily feed intake, average daily gain (ADG), and feed-to-gain ratio (F/G) were calculated accordingly. At the end of the trial, whole blood samples were collected for routine hematological analysis. Serum was separated and stored at −20 °C for subsequent determination of immune-related and tryptophan metabolism-related indices. In addition, three yaks were randomly selected from each treatment group for slaughter sampling. We acknowledge that *n* = 3 per group is relatively small for rumen tissue and scanning electron microscopy analysis, which is a potential limitation of this study. Dressing percentage was determined in these animals. Rumen contents were collected and used for subsequent analyses, and rumen tissue samples were obtained simultaneously for scanning electron microscopy observation.

### 2.3. Determination of Serum Biochemistry, Antioxidant Status, and Immune Parameters

Commercial enzyme-linked immunosorbent assay (ELISA) kits produced by Shanghai Kewei Biotechnology Co., Ltd. (Shanghai, China) were used to determine serum concentrations of immunoglobulin A (IgA), immunoglobulin G (IgG), and immunoglobulin M (IgM), as well as the levels of cytokines including interleukin-8 (IL-8), interleukin-2 (IL-2), and tumor necrosis factor-α (TNF-α). Meanwhile, the activities of superoxide dismutase (SOD) and glutathione peroxidase (GSH-Px) in serum were measured using assay kits purchased from Nanjing Jiancheng Bioengineering Institute (Nanjing, China). All experimental procedures were performed strictly in accordance with the standard protocols provided by the kit manufacturers.

### 2.4. Determination of Tryptophan and Its Metabolites in Serum

Serum tryptophan pathway metabolites were determined by UHPLC-QTRAP LC-ESI-MS/MS. Briefly, 33 metabolite standards were individually dissolved in 50% methanol to prepare stock solutions, which were then mixed and diluted with ultrapure water to obtain a series of working solutions. The isotopically labeled internal standard Trp-D5 was dissolved in methanol and further diluted with ultrapure water to 4000 ng/mL. Trp-D5 was used as the internal standard for tryptophan quantification, while the other 32 metabolites were quantified using external calibration curves prepared with authentic standards. For sample preparation, 100 μL of serum was mixed with 10 μL of internal standard working solution and 390 μL of methanol, followed by vortexing for 30 s, sonication at 5 °C and 40 kHz for 30 min, incubation at −20 °C for 30 min, and centrifugation at 13,000 rcf for 15 min at 4 °C. Then, 350 μL of the supernatant was collected, evaporated to dryness under nitrogen, and reconstituted in 70 μL of 1% acetonitrile aqueous solution containing 0.1% formic acid. After vortexing for 30 s, sonication at 5 °C and 40 kHz for 15 min, and centrifugation at 13,000 rcf for 15 min at 4 °C, the final supernatant was subjected to instrumental analysis. Chromatographic separation was performed on a Nexera Series LC-40 system equipped with an ACQUITY UPLC^®^ HSS T3 column (2.1 × 150 mm, 1.8 μm; Waters, Corporation, Milford, MA, USA) at 40 °C with an injection volume of 2 μL. The mobile phases consisted of 0.1% formic acid in water and 0.1% formic acid in acetonitrile. Mass spectrometric detection was conducted on an AB SCIEX QTRAP 7500+ instrument (SCIEX, Framingham, MA, USA) operated in both positive and negative ion modes with the following parameters: CUR 35, CAD medium, IS 5500/−4500, TEM 550, GS1 50, and GS2 50.

### 2.5. Rumen Microbiota Analysis Using Metagenomic Sequencing

Metagenomic sequencing was performed by Shanghai Majorbio Bio-Pharm Technology Co., Ltd. (Shanghai, China) on the Illumina NovaSeq platform (Illumina, Inc., San Diego, CA, USA). After quality filtering with fastp and removal of host and contaminant sequences using BWA (v0.7.9a), clean reads were assembled with MEGAHIT (v1.2.9), and contigs ≥ 300 bp were retained. Open reading frames were predicted using Prodigal/MetaGene (v2.6.3), and genes ≥ 100 bp were clustered with CD-HIT (v4.6.1) at 90% sequence identity and 90% coverage to generate a non-redundant gene catalog, in which the longest sequence of each cluster was used as the representative sequence. High-quality reads from each sample were then mapped to the non-redundant gene catalog using SOAP aligner (v2.21) at 95% sequence identity to estimate gene abundance.

### 2.6. Analysis of Rumen Short-Chain Fatty Acid Profiles

Rumen fluid samples were collected from yaks in each group and pretreated before analysis by gas chromatography–mass spectrometry (GC-MS) (Agilent Technologies Inc., Santa Clara, CA, USA). Chromatographic separation was performed under the conditions described in our previous study [14]. Reference standards were analyzed first to determine the retention times and peak areas of the target compounds, and the samples were then injected for analysis. Target compounds in the rumen fluid samples were identified by comparison with the retention times of the corresponding standards.

### 2.7. Scanning Electron Microscopic Observation of the Rumen Epithelium

Rumen tissue samples were rapidly collected, rinsed several times with phosphate-buffered saline (PBS) to remove surface debris and mucus, and immediately fixed in 3% glutaraldehyde. After fixation, the samples were rinsed three times with ultrapure water for 10 min each, post-fixed in 1% osmium tetroxide for 1–2 h, and rinsed again three times with ultrapure water for 10 min each. The samples were then dehydrated through a graded ethanol series of 30%, 50%, 70%, 90%, and 100%, with the 100% ethanol step repeated three times for 15 min each. After critical point drying, the samples were mounted on specimen stubs with conductive adhesive, sputter-coated with gold, and observed under a scanning electron microscope.

### 2.8. Data Analysis

All statistical analyses were performed using SPSS Statistics 26.0 (IBM Corp., Armonk, NY, USA). Data visualization was conducted with GraphPad Prism 8.0 (GraphPad Software Inc., San Diego, CA, USA). Measurement data are presented as the mean ± standard error of the mean (SEM). Differences among groups were analyzed by one-way analysis of variance (ANOVA), followed by Duncan’s multiple range test for multiple comparisons.

For microbiome analysis, raw sequencing data were processed with QIIME2 2020.6. Differential abundance analysis of taxonomic features among groups was performed using the linear discriminant analysis effect size (LEfSe) method, with a linear discriminant analysis (LDA) score > 2.0 and a significance level of the Kruskal–Wallis test set at α = 0.05. A probability value of *p* < 0.05 was considered statistically significant for all analyses in this study.

## 3. Results

### 3.1. Effects of WJPS on Growth Performance and Hematological Parameters in Yaks

As shown in Table 2, the ADG of yaks in all WJPS-supplemented groups showed an increasing trend compared with the CON group. Compared with the CON group, the F/G values were reduced by 40.4%, 53.5%, and 47.5% in the WJPS-L, WJPS-M, and WJPS-H groups, respectively. In addition, dressing percentage showed no significant difference among groups, although a slight numerical increase was observed in the WJPS-H group.

As shown in Table 3, compared with the CON group, the WJPS-L group showed significantly increased WBC, Lym, and RBC counts (*p* < 0.05). In the WJPS-M group, Lym and RBC counts were significantly higher than those in the CON group (*p* < 0.05). In the WJPS-H group, WBC and Lym counts were significantly increased compared with the CON group (*p* < 0.05). Neu was lower in the WJPS-M and WJPS-H groups than in the CON group. HGB, Mon and HCT showed no significant differences among the groups (*p* > 0.05).

### 3.2. Effects of WJPS on Serum Immune and Antioxidant Parameters in Yaks

As shown in Table 4, compared with the CON group, the levels of IL-2, IgG, and SOD were significantly increased in all WJPS treatment groups (*p* < 0.05). Compared with the CON group, IgG was significantly increased in all WJPS-treated groups (*p* < 0.05), whereas IgM was significantly increased mainly in the WJPS-L and WJPS-H groups (*p* < 0.05). Among them, the levels of IL-8 and GSH-Px were significantly elevated in the WJPS-L and WJPS-M groups (*p* < 0.05), whereas IL-8 and GSH-Px in the WJPS-H group showed an increasing trend but the differences were not statistically significant (*p* > 0.05). In addition, although the levels of TNF-α and IgA tended to increase in all WJPS treatment groups, the differences among groups were not statistically significant (*p* > 0.05).

### 3.3. Ultrastructure of Ruminal Epithelium and Microbial Colonization

**Figure 1:**
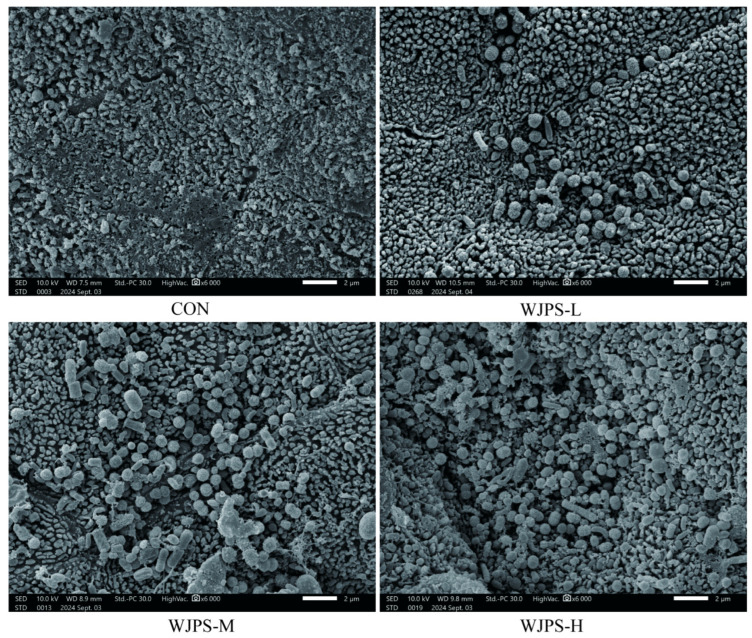
Ultrastructure of Ruminal Epithelium and Microbial Colonization. (*n* = 3).

As shown in Figure 1, scanning electron microscopy (×6000, scale bar = 2 μm) revealed that the ultrastructure of the ruminal mucosal epithelium remained intact in all four groups, with no evidence of epithelial cell shedding, erosion, or focal defects, indicating that the papillary epithelial barrier was well preserved. In the CON group, bacterial cells were observed on the epithelial surface, with cocci as the predominant morphotype showing a relatively uniform distribution. In the WJPS-L group, cocci were regularly distributed on the epithelial surface. The WJPS-M group showed a high colonization density, mainly composed of cocci, together with numerous clearly visible short rods interspersed to form a dense and continuous bacterial layer. In the WJPS-H group, cocci and short rods were present at a relatively balanced proportion, with a high colonization density and a more irregular distribution pattern. No abnormal bacterial morphology, fungal colonization, or inflammatory injury was observed in any group, suggesting that the experimental treatments did not impair the ruminal epithelial barrier. Overall, only physiological differences in microbial colonization patterns were observed among groups, providing a morphological basis for subsequent microecological analysis.

### 3.4. Effects of WJPS on Ruminal Short-Chain Fatty Acid Concentrations in Yaks

**Figure 2:**
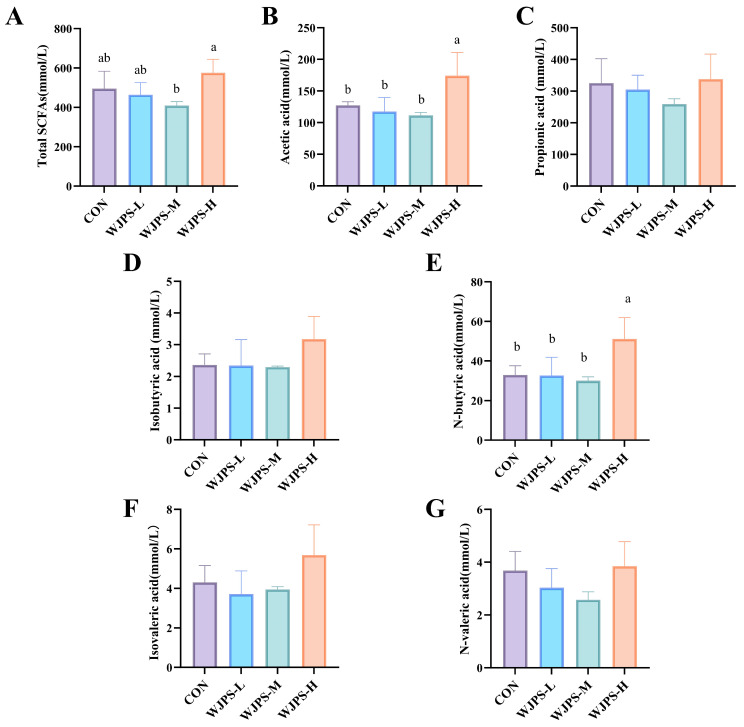
Effects of WJPS on ruminal short-chain fatty acid concentrations in yaks. (**A**) Total SCFAs; (**B**) Acetic acid; (**C**) Propionic acid; (**D**) Isobutyric acid; (**E**) N-Butyric acid; (**F**) Isovaleric acid; (**G**) N-Valeric acid; Means within a row with different superscripts are significantly different (*p* < 0.05, *n* = 3).

As shown in Figure 2, compared with the CON group, the total short-chain fatty acid content was decreased in the WJPS-L and WJPS-M groups, while it was increased in the WJPS-H group. In the WJPS-H group, the contents of butyrate and acetate were significantly increased (*p* < 0.05).

For other short-chain fatty acid components, the contents of propionate, isobutyrate, valerate and isovalerate were decreased in the WJPS-L and WJPS-M groups, but increased in the WJPS-H group. The differences in propionate, isobutyrate, valerate and isovalerate contents among all groups were not statistically significant (*p* > 0.05).

### 3.5. Effects of WJPS on Serum Tryptophan Metabolism in Yaks

**Figure 3:**
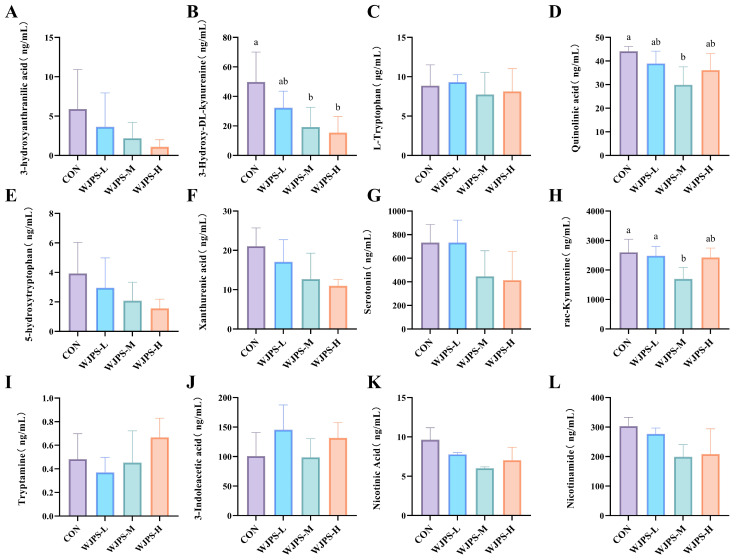
Effects of WJPS on Serum Tryptophan Metabolism in Yaks: (**A**) 3-hydroxyanthranilic acid; (**B**) 3-Hydroxy-DL-kynurenine; (**C**) L-Tryptophan; (**D**) Quinolinic acid; (**E**) 5-hydroxytryptophan; (**F**) Xanthurenic acid; (**G**) Serotonin; (**H**) rac-Kynurenine; (**I**) Tryptamine; (**J**) 3-indoleacetic acid; (**K**) Nicotinic Acid; (**L**) Nicotinamide. Means within a row with different superscripts are significantly different (*p* < 0.05, *n* = 3).

The kynurenine pathway, as a major route of tryptophan catabolism, is closely associated with host immune and inflammatory status. Therefore, changes in kynurenine-related metabolites were analyzed in the serum of yaks. As shown in Figure 3, 3-hydroxyanthranilic acid, 5-hydroxytryptophan, and xanthurenic acid within this pathway decreased in a dose-dependent manner with increasing dietary levels of WJPS. The level of 3-hydroxy-DL-kynurenine was significantly reduced in the WJPS-treated groups (*p* < 0.05), with the greatest decrease observed in the WJPS-H group. DL-kynurenine was significantly decreased in the WJPS-M and WJPS-H groups (*p* < 0.05), whereas no significant change was observed in the WJPS-L group. Quinolinic acid was significantly reduced in all WJPS treatment groups (*p* < 0.05). Regarding other tryptophan-related metabolites, L-tryptophan and serotonin showed a slight increase in the WJPS-L group but tended to decrease in the WJPS-M and WJPS-H groups. By contrast, tryptamine was decreased in the WJPS-L group and tended to increase in the WJPS-M and WJPS-H groups, although these changes were not statistically significant (*p* > 0.05). In the indole and nicotinic acid branches, 3-indolelactic acid was slightly increased in the WJPS-L and WJPS-H groups but decreased in the WJPS-M group. Nicotinic acid showed a decreasing trend in the WJPS-L and WJPS-M groups and a slight increase in the WJPS-H group. Nicotinamide also decreased in a dose-dependent manner in all WJPS-treated groups, although the differences were not statistically significant (*p* > 0.05). Overall, WJPS intervention altered several key metabolites involved in serum tryptophan metabolism in yaks. The reduction in quinolinic acid, a pro-inflammatory and neurotoxic metabolite, indicates suppression of the indoleamine 2,3-dioxygenase (IDO)/tryptophan 2,3-dioxygenase (TDO) pathway, thereby reducing inflammatory stress and supporting the observed elevation in IgG and IL-2.

### 3.6. Effects of WJPS on Ruminal Microbial Flora Composition in Yaks

**Figure 4:**
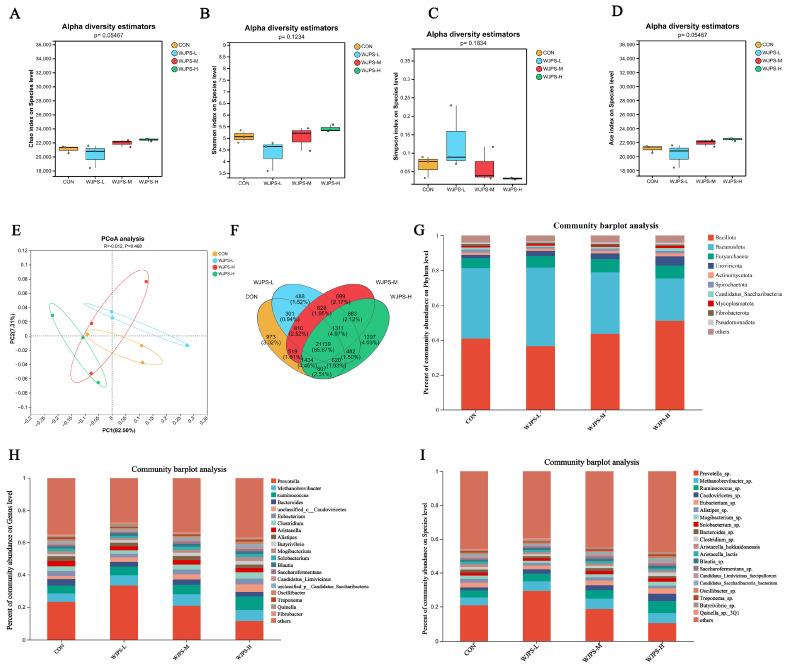
Effects of WJPS on the diversity and composition of yak rumen microbiota: (**A**) Chao index of rumen microbiota; (**B**) Shannon index of rumen microbiota; (**C**) Simpson index of rumen microbiota; (**D**) Ace index of rumen microbiota; (**E**) Principal coordinate analysis (PCoA) based on Bray–Curtis distance; (**F**) Venn diagram of shared and unique microbial species; (**G**) Community barplot analysis of microbial abundance at the phylum level; (**H**) Community barplot analysis of microbial abundance at the genus level; (**I**) Community barplot analysis of microbial abundance at the species level. Significant difference was defined as *p* < 0.05 (n = 8).

To investigate the effects of WJPS on the rumen microbial community in yaks, alpha diversity was first assessed. As shown in Figure 4A–D, compared with the CON group, the Chao, Shannon, and Ace indices were slightly increased in the WJPS-L group, whereas the Simpson index was slightly decreased, indicating a tendency toward increased microbial richness and diversity. In contrast, the WJPS-M and WJPS-H groups showed slight decreases in the Chao, Shannon, and Ace indices and a slight increase in the Simpson index, suggesting a shift in community structure. None of these alpha diversity indices differed significantly among the WJPS treatment groups (*p* > 0.05). However, the observed trends suggest that WJPS supplementation may still influence the richness and diversity of the rumen microbial community to some extent, particularly at different dietary levels.

Principal coordinate analysis based on Bray–Curtis distance (Figure 4E) showed a certain degree of separation among the CON, WJPS-L, WJPS-M, and WJPS-H groups, with the greatest separation observed between the WJPS-H and CON groups. Nevertheless, partial overlap remained among groups, indicating that although WJPS supplementation tended to affect the overall rumen microbial structure, the differences were not statistically significant (*p* > 0.05). Therefore, WJPS tended to reshape the rumen microbial community rather than significantly change the community structure.

The Venn diagram of species composition (Figure 4F) showed that the four groups shared a large number of taxa, together with a limited number of unique taxa, among which the WJPS-H group contained relatively more unique taxa. At the phylum level (Figure 4G), the dominant phyla were *Bacillota*, *Bacteroidota*, *Euryarchaeota*, and *Uroviricota*, with a total relative abundance exceeding 80%. Compared with the CON group, the relative abundance of *Bacteroidota* decreased, whereas that of *Bacillota* increased in the WJPS-H group. At the genus level (Figure 4H), *Prevotella*, *Ruminococcus*, and *Methanobrevibacter* were the dominant genera in all groups. Compared with the CON group, the abundance of *Prevotella* decreased, whereas that of *Ruminococcus* increased in the WJPS-H group. At the species level (Figure 4I), the relative abundance of *Prevotella* sp. was markedly reduced in the WJPS-H group, whereas those of *Ruminococcus* sp. and *Caudoviricetes* sp. were increased, which was generally consistent with the changes observed at the genus level.

Overall, WJPS supplementation tended to reshape the rumen microbial community in yaks, particularly at the high supplementation level, although its effects on overall alpha diversity were limited.

**Figure 5:**
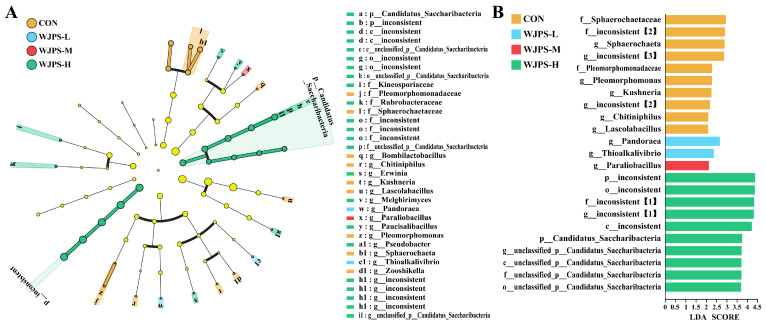
The rumen microbiota differences between the control group and the WJPS groups of yaks were identified by LEfSe analysis: (**A**) Bar plot showing taxa with significant differences identified by LEfSe; (**B**) Cladogram illustrating the phylogenetic distribution of differentially abundant taxa. Bracketed numbers after taxonomic labels indicate different taxa sharing the same unresolved annotation. Significant difference was defined as *p* < 0.05 (*n* = 8).

LEfSe analysis with an LDA score threshold of 2 was performed to identify differential microbial taxa among the control group CON and the WJPS-supplemented groups. As shown in Figure 5A, the CON group was enriched in taxa including *f_Sphaerochaetaceae* and *g_Sphaerochaeta*. In the WJPS-L group, only *g_Pandorea* and *g_Thioalkalivibrio* were enriched at the genus level. In contrast, the WJPS-H group showed enrichment of *p_Candidatus Saccharibacteria* and related unclassified taxa across multiple taxonomic levels, from phylum to genus.

The LDA score histogram further quantified these differences. As shown in Figure 5B, taxa such as *f_Sphaerochaetaceae* and *g_Sphaerochaeta* in the CON group had relatively high LDA scores. In the WJPS-L group, only *g_Pandorea* and *g_Thioalkalivibrio* showed LDA scores greater than 2. By contrast, multiple taxa related to *Candidatus Saccharibacteria* in the WJPS-H group exhibited LDA scores above 2, indicating a stronger differential enrichment effect.

These results suggest that the CON group was mainly characterized by *Sphaerochaeta*-related taxa, whereas the WJPS-L group showed enrichment of only a limited number of specific genera. In comparison, the WJPS-H group exhibited broader effects on the rumen microbial community, particularly through enrichment of taxa associated with *Candidatus Saccharibacteria*.

### 3.7. Correlation Analysis

**Figure 6:**
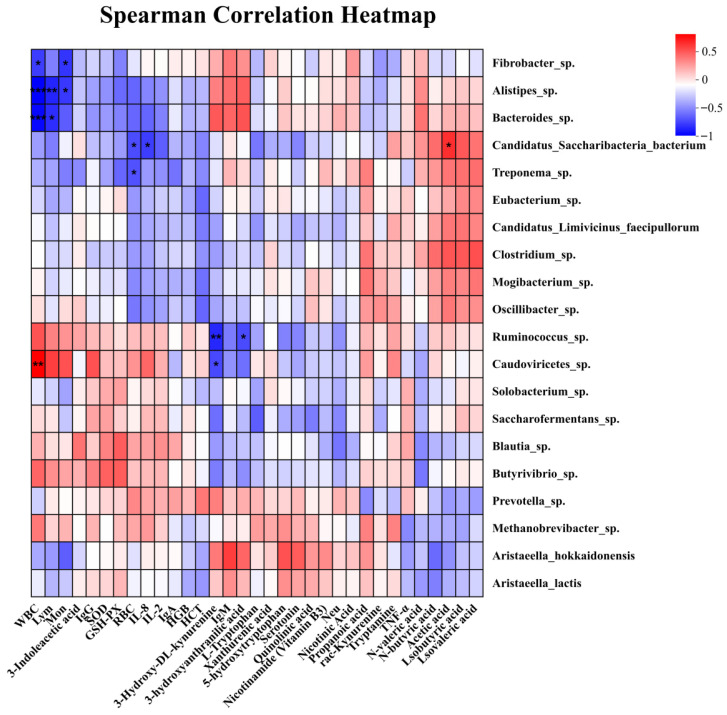
Spearman’s correlation between yak rumen microbiota and growth performance, serum physiological and biochemical indices, as well as tryptophan metabolism. Red represents a positive correlation and blue represents a negative correlation. The color intensity is proportional to the magnitude of the Spearman correlation coefficient. * *p* < 0.05 indicates a significant difference; ** *p* < 0.01 indicates a highly significant difference. *** *p* < 0.001 indicates an extremely significant difference.

Correlation analysis was performed between the top 20 dominant species in the rumen microbiota and hematological parameters, antioxidant status, immune indices, serum tryptophan metabolites, and rumen short-chain fatty acids. As shown in Figure 6, *Fibrobacter* sp. was negatively correlated with WBC (R = −0.657, *p* < 0.05) and Mon (R = −0.685, *p* < 0.05). *Alistipes* sp. was negatively correlated with WBC (R = −0.846, *p* < 0.05), Lym (R = −0.734, *p* < 0.05), and Mon (R = −0.671, *p* < 0.05). *Bacteroides* sp. was negatively correlated with WBC (R = −0.825, *p* < 0.05) and Lym (R = −0.706, *p* < 0.05). *Candidatus Saccharibacteria bacterium* was negatively correlated with RBC (R = −0.608, *p* < 0.05) and IL-8 (R = −0.657, *p* < 0.05), but positively correlated with acetic acid (R = 0.594, *p* < 0.05). *Ruminococcus* sp. was negatively correlated with 3-hydroxy-DL-kynurenine (R = −0.727, *p* < 0.05) and 3-hydroxyanthranilic acid (R = −0.615, *p* < 0.05). *Caudoviricetes* sp. was negatively correlated with 3-hydroxy-DL-kynurenine (R = −0.622, *p* < 0.05) and positively correlated with WBC (R = 0.741, *p* < 0.05). In addition, other dominant species also showed varying degrees of association with the measured indices, although these correlations did not reach statistical significance.

## 4. Discussion

The present study demonstrated that dietary supplementation with WJPS, particularly at 2.0%, improved growth performance and host physiological status in yaks, accompanied by coordinated changes in rumen microbial composition, SCFA production, and tryptophan metabolism. Although the 2.0% inclusion level is relatively high, the significant improvement in feed efficiency, immune function, and overall health can offset the cost of herbal additives in practical yak production. Future studies can explore lower effective doses to balance efficacy and economic benefits for large-scale farming. These findings suggest that WJPS may exert its beneficial effects not only through nutritional supplementation, but also through modulation of the rumen microecological environment and microbe–host metabolic interactions. As a characteristic ruminant species adapted to alpine regions, the yak is especially vulnerable to nutritional limitation and microbial imbalance under harsh environmental conditions. Therefore, the improvement induced by WJPS is of particular relevance for maintaining rumen homeostasis and promoting health in plateau production systems.

### 4.1. Regulatory Effects of WJPS on Growth Performance in Yaks

The present study showed that WJPS supplementation improved growth performance in yaks, as reflected by increased ADG and decreased F/G, with the most pronounced effect observed at the 2.0% supplementation level. Previous studies have reported that compound feed additives containing qi-tonifying Chinese herbs, such as *Astragalus membranaceus* and *Codonopsis pilosula*, can increase ADG and reduce F/G in beef cattle, mainly by improving nutrient digestion and utilization efficiency [15,16]. The present findings are consistent with these reports and further suggest that WJPS may enhance growth performance in yaks by improving feed utilization. To further elucidate this effect, future studies will incorporate apparent digestibility trials to more directly assess the influence of WJPS on nutrient digestion, absorption, and utilization in yaks.

In contrast, WJPS supplementation did not significantly affect dressing percentage. This may be related to the distinctive physiological characteristics of yaks as a high-altitude-adapted species. Under plateau conditions, yaks tend to allocate nutrients toward survival, thermoregulation, and adaptation to hypoxia rather than toward carcass deposition alone. Therefore, the effects of Chinese herbal feed additives in yaks may be reflected more in improved growth efficiency and physiological status than in slaughter traits [17]. From this perspective, the growth-promoting effect of WJPS appears to be more consistent with the goal of healthy and efficient yak production than with a simple fattening effect.

### 4.2. Regulatory Mechanism of WJPS on Immunity and Antioxidant Capacity in Yaks

WJPS supplementation significantly increased several key hematological and immune indices in yaks, including WBC and Lym, and also elevated the serum levels of IL-2, IgG, and SOD. Notably, the low- and medium-dose groups showed more pronounced effects on IL-8 and GSH-Px. Previous studies have shown that polysaccharides from *Astragalus membranaceus* and saponins from *Codonopsis pilosula* can enhance macrophage and lymphocyte activity and promote the expression of immune-related factors such as IgG and IL-2 [18,19,20]. In addition, polyphenolic components from *Crataegus pinnatifida* and *Citrus reticulata* exhibit marked antioxidant activity and can increase the activities of antioxidant enzymes, including SOD and GSH-Px [21,22]. Therefore, the improvement in antioxidant and immune status observed in the present study may be attributable to the synergistic effects of the herbal components in WJPS.

High-altitude hypoxia is known to aggravate oxidative stress and impair immune function in ruminants [23]. In the present study, all doses of WJPS significantly increased SOD levels, suggesting that WJPS effectively alleviated oxidative stress in yaks and may be particularly beneficial under plateau conditions. In contrast, WJPS had no significant effect on TNF-α, indicating that it enhanced immune function without inducing excessive inflammatory activation. This finding suggests that WJPS may help maintain a balance between immune enhancement and inflammatory homeostasis. Compared with reports showing that some Chinese herbal compounds may overstimulate immune responses and elevate pro-inflammatory factors [24,25] the present results indicate that WJPS exerts a relatively moderate and coordinated regulatory effect.

### 4.3. Regulatory Patterns and Microecological Significance of WJPS on Rumen Microbiota and SCFAs in Yaks

Scanning electron microscopy showed that WJPS did not cause detectable structural damage to the rumen epithelium, while differences in surface-associated microbial colonization patterns were observed among groups. This suggests that WJPS may modulate the rumen microecological environment without disrupting epithelial integrity, providing morphological support for the subsequent microbiota analysis. As these electron microscopy observations were performed on randomly selected animals, future studies with expanded tissue sampling would help provide more comprehensive morphological evidence for the effects of WJPS on rumen epithelial structure and surface-associated microbial colonization.

The rumen microbiota of yaks was dominated by *Bacillota* and *Bacteroidota* [26,27], which is consistent with our findings and with the core rumen microbial structure reported in other ruminants, including dairy cows and beef cattle [17,28]. Notably, high-dose WJPS increased the relative abundance of *Bacillota* while decreasing that of *Bacteroidota*, a pattern generally consistent with previous reports on the modulatory effects of Chinese herbal compounds on the rumen microbiota of ruminants [29]. Because the *Bacillota*/*Bacteroidota* ratio is closely related to nutrient utilization [30], this shift may indicate improved fermentation efficiency. This interpretation is supported by the increased total SCFAs, acetate, and n-butyrate observed in the high-dose group. Acetate is a major substrate for energy metabolism in ruminants [31], whereas n-butyrate supports epithelial function and barrier maintenance [32,33], suggesting that these fermentation changes may contribute to improved growth and host physiological status. WJPS-L may cause transient microbial adaptive stress and slightly suppress rumen fermentation, while a sufficiently high dosage remodels rumen microbiota structure, optimizes substrate utilization, and thereby enhances short-chain fatty acid production and fermentation function.

At the genus level, high-dose WJPS was associated with a decreased abundance of *Prevotella* and an increased abundance of *Ruminococcus* [34]. Although this pattern differs somewhat from reports on some other Chinese herbal compounds, it may reflect differences in dietary substrate conditions. *Prevotella* is generally associated with protein and starch utilization, whereas *Ruminococcus* is more closely linked to fiber degradation [35]. These findings suggest that WJPS may improve the overall efficiency of ruminal nutrient utilization by modulating microbial substrate utilization patterns rather than selectively affecting a single type of substrate. This observation provides additional insight into how Chinese herbal compounds regulate the rumen microbiota of ruminants under different dietary conditions.

In addition, the high-dose WJPS group was enriched in taxa related to *Candidatus_Saccharibacteria* [36], a group of uncultured microorganisms associated with the rumen ecosystem. This result suggests that WJPS may also influence less characterized rumen-associated microbial taxa. To our knowledge, few studies have examined the response of this group to Chinese herbal compound supplementation. Therefore, the present findings expand the current understanding of how Chinese herbal compounds may influence uncultured rumen-associated microorganisms.

In the present study, the effect of WJPS on SCFAs showed a dose-dependent pattern, with enhancement at the high dose but slight reduction at the low and medium doses, which was generally consistent with the observed changes in microbial community structure. The low- and medium-dose groups showed slightly increased microbial richness, whereas the high-dose group exhibited relatively better community evenness, suggesting that remodeling of the rumen microbiota may require a sufficient supplementation level of WJPS. At lower doses, the microbial community may still have been in an adaptive state, whereas at the high dose, the community structure appeared to be more stable and metabolically active. Additionally, the relatively low acetate proportion observed in this study can be explained by the 40:60 forage-to-concentrate diet: although the diet contained a majority of forage, the 40% concentrate still shifted fermentation toward greater propionate production, reducing the molar percentage of acetate, which is consistent with previous findings that moderate increases in dietary concentrate decrease acetate and the acetate: propionate ratio compared with higher forage diets [37]. These dose-dependent microbial responses and fermentation patterns provide useful support for the practical application of WJPS in yak production.

### 4.4. Regulation of Tryptophan Metabolism by WJPS and the Core Mechanism of Microbiota–Host Interaction in Yaks

The kynurenine pathway of tryptophan metabolism is increasingly recognized as an important interface linking rumen microbiota with host immune metabolism [38]. In the present study, WJPS reduced the levels of several key metabolites in this pathway, particularly 3-hydroxy-DL-kynurenine and quinolinic acid. This finding is noteworthy because previous studies have shown that excessive activation of the kynurenine pathway and accumulation of its downstream metabolites can suppress T-cell proliferation, impair immune function, and aggravate oxidative stress [39,40]. Therefore, the inhibitory effect of WJPS on these key metabolites may partly explain its beneficial effects on immune and antioxidant status in yaks.

Correlation analysis further suggested a close association among WJPS supplementation, rumen microbial changes, tryptophan metabolism, and host physiological responses. Specifically, Ruminococcus and Caudoviricetes were significantly negatively correlated with kynurenine pathway metabolites, whereas Alistipes, Bacteroides, and several other taxa were negatively correlated with WBC and Lym. These results suggest that WJPS may influence host immune function, at least in part, through regulation of core rumen microorganisms and their downstream metabolic pathways. Of particular interest, Caudoviricetes represent rumen-associated bacteriophages, and their enrichment may contribute to maintaining microbial ecological balance by modulating bacterial community structure [41,42,43,44]. This observation extends the current understanding of microbiota–host interactions and suggests that phage-associated changes may also participate in the metabolic and immune effects of Chinese herbal compound supplementation.

Notably, WJPS had no significant effect on tryptophan itself or on its major derivatives such as L-tryptophan and 5-hydroxytryptamine, but mainly affected the kynurenine branch. This pattern suggests that the regulatory effect of WJPS on tryptophan metabolism may be relatively specific, in that it does not broadly disturb total tryptophan availability but instead shifts metabolic flux away from the production of potentially harmful downstream metabolites. Such a pattern may be advantageous for maintaining metabolic homeostasis while limiting excessive accumulation of immunosuppressive or pro-oxidative products. Moreover, the regulatory effect of WJPS on tryptophan metabolism was most evident in the high-dose group, which was consistent with the trends observed for rumen microbial structure, SCFA production, and growth performance. Taken together, these findings indicate that the effects of WJPS in yaks are more likely to reflect coordinated regulation across multiple physiological and metabolic layers rather than isolated changes in individual indicators.

## 5. Conclusions

Dietary WJPS supplementation tended to improve growth performance and significantly enhanced antioxidant capacity and immune status in yaks, with 2.0% showing the most favorable overall effect. The most definitive impact of WJPS was on the immune-metabolic axis, including improved rumen SCFA profiles and regulated kynurenine pathway metabolism. These benefits were accompanied by tendential modulation of rumen microbial composition, SCFA production, and tryptophan metabolism, without detectable damage to the rumen epithelial barrier. The findings suggest that WJPS may promote yak health through regulation of the rumen microecological environment and host metabolic–immune homeostasis. Therefore, WJPS has potential as a natural feed additive for plateau yak production and provides new insight into the application of Chinese herbal compounds in rumen microecological regulation.

## Figures and Tables

**Table 1:** Composition and nutritional levels of yak concentrate (DM basis, %).

Items	Concentrate
**Ingredients**	
Corn	55
Soybean meal	15
Rapeseed meal	15
*Hordeum vulgare* var. *coeleste* Linnaeus	13
NaHCO_3_	1
NaCl	1
Total	100
**Nutrition Level ^1^**	
DM	85.745
CP	20.617
Ether extract	3.945
NDF	7.83
ADF	13.781
Crude ash	7.723
Ca	0.2802
P	0.4986
Gross energy/(MJ/kg)	11.447

^1^ Nutrient levels were calculated values. Abbreviations: DM, dry matter; CP, crude protein; NDF, neutral detergent fiber; ADF, acid detergent fiber.

**Table 2:** Effects of WJPS on growth performance.

Items	CON	WJPS-L	WJPS-M	WJPS-H	*p*-Value
Dressing Percentage (%)	54.31 ± 2.86	52.17 ± 2.16	54.02 ± 3.12	61.27 ± 10	0.272
ADG (kg/d)	0.35 ± 0.04	0.73 ± 0.39	0.76 ± 0.18	0.71 ± 0.19	0.084
F/G	16.15 ± 1.77 ^a^	9.62 ± 5.09 ^b^	7.5 ± 1.46 ^b^	8.47 ± 1.99 ^b^	0.006

Means within a row with different superscripts are significantly different (*p* < 0.05, n = 8). Abbreviations: CON, control group; WJPS-L, 0.5% WJPS group; WJPS-M, 1.0% WJPS group; WJPS-H, 2.0% WJPS group; ADG, average daily gain; F/G, feed-to-gain ratio.

**Table 3:** Hematological parameters in yaks.

Items	CON	WJPS-L	WJPS-M	WJPS-H	*p*-Value
WBC (10^9^/L)	7.11 ± 0.58 ^b^	8.5 ± 0.86 ^a^	7.99 ± 0.52 ^ab^	8.23 ± 0.88 ^a^	0.02
Neu (%)	3.1 ± 0.4	2.81 ± 0.49	2.52 ± 0.22	2.5 ± 0.46	0.061
Lym (%)	3.37 ± 0.27 ^b^	5.04 ± 0.46 ^a^	4.43 ± 0.56 ^a^	4.62 ± 0.75 ^a^	0.001
Mon (10^9^/L)	0.53 ± 0.19	0.59 ± 0.11	0.62 ± 0.25	0.59 ± 0.17	0.886
RBC (10^12^/L)	7.17 ± 0.73 ^c^	8.55 ± 0.28 ^a^	8.08 ± 0.58 ^ab^	7.69 ± 0.24 ^bc^	0.001
HGB (g/L)	148 ± 10.49	158.67 ± 6.77	147.5 ± 8.83	149.5 ± 4.89	0.084
HCT (%)	40.65 ± 2.82	43.52 ± 1.42	40.7 ± 2.39	40.9 ± 1.64	0.088

Means within a row with different superscripts are significantly different (*p* < 0.05, *n* = 8). Abbreviations: WBC, white blood cell; Neu, neutrophil; Lym, lymphocyte; Mon, monocyte; RBC, red blood cell; HGB, hemoglobin; HCT, hematocrit.

**Table 4:** Serum Immune and Antioxidant Indices in Yaks.

Items	CON	WJPS-L	WJPS-M	WJPS-H	*p*-Value
TNF-α (pg/mL)	14.09 ± 1.89	16.54 ± 3.25	16.52 ± 0.71	16.78 ± 5.32	0.462
IL-8 (pg/mL)	74.03 ± 17.2 ^b^	101.74 ± 6.17 ^a^	100.24 ± 7.69 ^a^	88.04 ± 18.04 ^ab^	0.006
IL-2 (pg/mL)	92.96 ± 18.2 ^d^	153.36 ± 12.99 ^a^	136.25 ± 7.94 ^c^	117.32 ± 13.62 ^b^	0.001
IgG (mg/mL)	8.69 ± 1.51 ^b^	11.21 ± 0.56 ^a^	11.16 ± 1.00 ^a^	10.02 ± 1.17 ^a^	0.002
IgA (mg/mL)	247.25 ± 16.19	269.93 ± 7.28	258.76 ± 27.19	248.58 ± 36.15	0.361
IgM (mg/mL)	466.45 ± 79.63 ^c^	612.27 ± 45.72 ^a^	532.27 ± 71.54 ^bc^	557.22 ± 45.68 ^ab^	0.006
GSH-Px (U/mL)	86.36 ± 40.66 ^b^	167.1 ± 41.73 ^a^	171.69 ± 64.68 ^a^	132.59 ± 58.72 ^ab^	0.038
SOD (U/mL)	50.14 ± 3.92 ^c^	86.62 ± 3.05 ^a^	82.93 ± 8.21 ^ab^	77.4 ± 3.47 ^b^	0.001

Means within a row with different superscripts are significantly different (*p* < 0.05, *n* = 8). Abbreviations: TNF-α, tumor necrosis factor-α; IL, interleukin; IgG, immunoglobulin G; IgA, immunoglobulin A; IgM, immunoglobulin M; GSH-Px, glutathione peroxidase; SOD, superoxide dismutase.

## Data Availability

The raw metagenomic sequencing reads were deposited into the NCBI Sequence Read Archive (SRA) database (Accession Number: PRJNA1452561). The other datasets used and analysed during the current study are available from the corresponding author on reasonable request.

## Abbreviations

The following abbreviations are used in this manuscript:

ADG	Average daily gain
ADF	Acid detergent fiber
ANOVA	Analysis of variance
CAD	Collision gas
CON	Control group
CP	Crude protein
DM	Dry matter
ELISA	Enzyme-linked immunosorbent assay
F/G	Feed-to-gain ratio
GC-MS	Gas chromatography–mass spectrometry
GSH-Px	Glutathione peroxidase
HCT	Hematocrit
HGB	Hemoglobin
IDO	Indoleamine 2,3-dioxygenase
IgA	Immunoglobulin A
IgG	Immunoglobulin G
IgM	Immunoglobulin M
IL	Interleukin
LDA	Linear discriminant analysis
LEfSe	Linear discriminant analysis effect size
Lym	Lymphocyte
Mon	Monocyte
NDF	Neutral detergent fiber
Neu	Neutrophil
PBS	Phosphate-buffered saline
PCoA	Principal coordinate analysis
RBC	Red blood cell
SCFAs	Short-chain fatty acids
SEM	Standard error of the mean
SOD	Superoxide dismutase
SRA	Sequence Read Archive
TCM	Traditional Chinese medicine
TDO	Tryptophan 2,3-dioxygenase
TNF-α	Tumor necrosis factor-α
Trp-D5	Deuterated tryptophan
WBC	White blood cell
WJPS	Wuwei Jianpi San
WJPS-H	High-dose group supplemented with 2.0% WJPS
WJPS-L	Low-dose group supplemented with 0.5% WJPS
WJPS-M	Medium-dose group supplemented with 1.0% WJPS
